# 

**Published:** 1875-08

**Authors:** 


### Table of Contents.

### ORIGINAL COMMUNICATIONS.

MEMBRANOUS DYSMENORRHŒA. *By Robert Battey, M.D., Atlanta, Ga.* ... 257
COLD DRY AIR as a therapeutic measure in Pulmonary Diseases. *By C. H. Harris, M.D., Cedar Town, Ga.* ... 262
ADHERENT PLACENTA—Case. *By B. R. Rives, M.D., Adams' Station, Ga.* 265
INTESTINAL OBSTRUCTION removed by position, distensile enema and gas. *By A. B. Copeland, M.D., Valley Plains, Ga.* ... 269

### ATLANTA ACADEMY OF MEDICINE.

Non-emetic doses of Ipecacuanha in Dysentery, 270—Uterine Fibrous Polypus, does it return? Treatment by local use of tinct. iron, 271 Reduction of Paraphymosis, alarming effects from Squibb's ether, 272—Anomalous case of Myalgia (?) of leg—Microscopical report on dysmenorrhœal membrane, 273—Membranous Dysmenorrhœa, 274 Dr. Alexander's new Pessary ... 275

### GEORGIA MEDICAL SOCIETY, SAVANNAH.

Herpes—Erysipelas 276—Hemorrhoids, discussion ... 277

### OUR EXCHANGES.

Dugas' Sign of Dislocations of the Shoulder-joint ... 278
Newspaper Charlatanism ... 285
Somnambulist on Guard ... 286
Elixir Iodo-Bromide Calcium Compound ... 287
Damiana New Aphrodisiac ... 291
Question of Quacks and Diplomas ... 292
Argumentium ad Hominem ...
Popular Health Almanac ... 295
Dr. J. Marion Sims ...
Oleomargarine, or Suet Butter ... 296
Tinct. Iron in Nasal Polypus ... 297
"A Lost Art" ... 298
Medical Education and Legislation ... 299
Medical Protective Association of New Orleans ... 301
Salicylic Acid as a Disinfectant ...
Novel Treatment of Vomiting of Pregnancy ... 302
Women in Ohio Medical Society ... 303
What's in a Name? ... 304
What is Menstruation? ... 305
Some Wise Men of Gotham ... 306
Natural History of Acute Dysentery ... 307
Form of Lightning Rods ... 309
Strangulated Hernia, new treatment ... 310
Render unto Cæsar, etc ... 311

### BIBLIOGRAPHICAL.

Ziemssen's Cyclopædia of Practice ... 311
Chambers' Manual of Diet ...
Campbell on uterine Displacements ... 313
Pamphlets ... 314

### EDITORIAL.

Pharmacy in Georgia ... 314
An Honest Confession ... 316
South Georgia Medical Society ... 317
Physiology of Menstruation ... 318
Child Killed by a Chicken ... 319
Miscellaneous Notes ... 320

